# Effect of Organic Farming and Agricultural Abandonment on Beneficial Arthropod Communities Associated with Olive Groves in Western Spain: Implications for *Bactrocera oleae* Management

**DOI:** 10.3390/insects13010048

**Published:** 2022-01-01

**Authors:** Víctor de Paz, Estefanía Tobajas, Natalia Rosas-Ramos, José Tormos, Josep Daniel Asís, Laura Baños-Picón

**Affiliations:** Departamento de Biología Animal, Ecología, Parasitología, Edafología y Química Agrícola, Facultad de Farmacia, Campus Miguel de Unamuno s/n, Universidad de Salamanca, 37007 Salamanca, Spain; estefaniatob@usal.es (E.T.); nataliarosasr@usal.es (N.R.-R.); tormos@usal.es (J.T.); asis@usal.es (J.D.A.); lbanos@usal.es (L.B.-P.)

**Keywords:** abandonment, organic farming, traditional olive groves, *Bactrocera oleae*, parasitoids, spiders, staphylinids

## Abstract

**Simple Summary:**

Olive cultivation has been extremely relevant in the Mediterranean area for centuries, creating traditional landscapes with high cultural and biodiversity values. However, in recent decades, these landscapes have been affected by two processes. On the one hand, the most productive areas have undergone significant intensification, with greater input of agrochemicals and a much higher tree density; on the other hand, marginal areas, with lower production, are being progressively abandoned. While more attention has been paid to intensification effects, few studies have considered the consequences of olive grove abandonment. In our study, we analyzed how abandonment and management regimes (organic or traditional) affected the main olive pest (*Bactrocera oleae*) and different groups of natural enemies in olive groves established near the border between Spain and Portugal. Our results showed that abandoned and managed olive groves had different, but similarly rich and diverse, communities of natural enemies, highlighting the complementary role that these two habitats play at the landscape scale. Moreover, abandoned groves may not be acting as a reservoir for the olive fly. To prevent land abandonment from continuing, measures such as organic farming or agritourism, which have been implemented in the studied area, could be effective.

**Abstract:**

Agricultural abandonment and intensification are among the main land-use changes in Europe. Along with these processes, different proposals have been developed to counteract the negative effects derived from agricultural intensification, including organic management. In this context, we aimed to determine how organic management and farmland abandonment affect *Bactrocera oleae* and its main groups of natural enemies: hymenopteran parasitoids, spiders, ants, carabids, and staphylinids. Between May and October 2018, four samplings were carried out in nine olive groves (three under organic management, three under traditional management, and three abandoned) in a rural area on the border between Spain and Portugal (Salamanca, Western Spain). Our results suggested differences between the natural enemy community composition of abandoned and organic groves, with slightly higher levels of richness and abundance in abandoned groves. We found no differences between organic and traditional groves. The managed olive groves sustained a different natural enemy community but were similarly rich and diverse compared with the more complex abandoned groves, with the latter not acting as a reservoir of *B. oleae* in our study area. Both systems may provide complementary habitats; however, further abandonment could cause a reduction in heterogeneity at the landscape scale and, consequently, a biodiversity loss.

## 1. Introduction

Land-use change is the main driver of biodiversity loss worldwide, with the expansion and intensification of agriculture—characterized by an increased input of synthetic pesticides, herbicides, and fertilizers—being the prime cause of insect population declines [1,2,3]. Biodiversity loss can negatively affect arthropod-mediated ecosystem services, such as pest control by natural enemies [4,5]. To mitigate the negative effects of agricultural intensification, different strategies have been developed, including organic management, in which synthetic pesticides, herbicides, and inorganic fertilizers are avoided [6]. Although there has been considerable controversy regarding the ability of organic management to support greater biodiversity, it has been consistently demonstrated that organic farming increases the richness and abundance of arthropods and other groups [4,6,7]. However, the positive effect of organic farming is highly dependent on the taxon and crop evaluated, and it is greater in annual crops and intensified landscapes [6,7]. Therefore, the landscape context modulates local biodiversity responses to organic farming, as predicted by the *intermediate landscape complexity hypothesis* [8]. This hypothesis states that in both cleared (<1% of non-crop habitat) and complex landscapes (>20% of non-crop habitat), only small positive responses to local agri-environmental management (such as organic farming) can be expected because of poor species pools and high immigration from semi-natural habitats, respectively, and that only simple landscapes, with intermediate levels of complexity, respond positively to agri-environmental schemes [9]. Moreover, increased biodiversity in organic farming may not happen in permanent crops, such as fruit orchards or vineyards, that present low disturbance levels, and the further reduction in the disturbance intensity of organic farming might not result in greater biodiversity [10]. This is explained by the *intermediate disturbance hypothesis*, which predicts higher diversity levels at intermediate levels of disturbance, with both slightly and highly disturbed systems harboring less biodiversity [11].

While agricultural intensification usually takes place on fertile soils, in marginal areas with traditional non-intensive farming systems, there is an ongoing process of farmland abandonment [12]. The consequences of the abandonment of traditional crops vary depending on the geographical region, scale, and taxa [13]. In Europe, traditional farming systems have existed for centuries and constitute high-nature-value systems [14], characterized by a low input of pesticides and synthetic fertilizers, with low levels of mechanization and high associated biodiversity, which are currently threatened by land abandonment [15,16,17]. In fact, Queiroz et al. [17], reviewing farmland abandonment effects on biodiversity, found that in Europe, most studies revealed negative effects. However, the majority of the studies that assessed farmland abandonment effects on arthropods were carried out in grasslands and annual crops in Central and Northern Europe.

In the Mediterranean Basin, a review by Plieninger et al. [18] revealed slightly positive effects of farmland abandonment on biodiversity, although their results were highly heterogeneous, and only four cases related to arthropods in permanent crops were included. They also found a decline in species richness after an abandonment period of fifty or more years that, they suggest, “may indicate that exclusion processes eventually follow colonization processes in many of the case studies.” Actually, many studies found higher biodiversity levels in the early stages of farmland abandonment, which tend to decrease as plant succession progresses [19,20,21,22]. In the early stages of farmland abandonment, plant diversity reaches its maximum, with herbaceous plants and scrubs coexisting, which results in habitats with high vegetation complexity [19,23]. Vegetation complexity, measured as structural and chemical complexity, enhances arthropod abundance and diversity by providing more microhabitats and resources, as well as diverse plant volatiles [20,24]. In the late stages of farmland abandonment, scrub and tree species of the surrounding vegetation become dominant, excluding open-habitat species, and reduce heterogeneity at a landscape scale, resulting in reduced biodiversity levels [13].

More work is needed to disentangle the effects of farmland abandonment on arthropod communities, especially in permanent crops, which are underrepresented in the bibliography, as well as in the Mediterranean Basin considering that it is one of the world’s diversity hotspots [25] and also an area where land abandonment is prevalent [26]. In this region, landscapes have been shaped by humans for millennia, creating different cultural landscapes that form the identity of the Mediterranean [27]. Such is the case of olive (*Olea europaea* L.)-dominated landscapes, where olive cultivation has been taking place since the Roman Age [28]. Within the Mediterranean region, Spain, with a production of almost 1.8 tons of olive oil and 10 tons of olives, is the largest producer [29]. Olive grove yields can be affected by a variety of pests, with the olive fruit fly, *Bactrocera oleae* (Rossi, 1790), being the most relevant [30]. *Bactrocera oleae* is attacked by a variety of natural enemies and it produces between three and five generations per year in the Mediterranean area, starting in early spring [31,32]. The adult flies oviposit in olives, where the larvae are vulnerable to hymenopteran parasitoids, and after completing their development, the larvae leave the fruits to pupate in the ground, where they are again exposed to generalist predators, mainly spiders, carabids and staphylinids, and ants [33,34].

Despite the importance of the olive groves in the Mediterranean region and specifically in Spain, studies that evaluated the effect of the abandonment of olive groves on the associated arthropod biodiversity are scarce (but see [35] for bees, [36] for isopods, and [37] for butterflies), and to our knowledge, no studies have evaluated the effect of olive grove abandonment on *B. oleae* and the arthropod groups that include its most important natural enemies, especially in traditional landscapes with different management regimes. In this context, we set out to determine (i) the possible effect of organic management and farmland abandonment on *B. oleae* and the structure and composition of the natural enemy community in traditional olive groves in a complex landscape; (ii) the differences in richness, abundance, and diversity of natural enemies between organic and abandoned olive groves; and (iii) the response of the dominant natural enemy families and *B. oleae* to olive grove abandonment. Considering the *intermediate disturbance hypothesis* and the *intermediate landscape complexity* *hypothesis*, we hypothesized that *B. oleae* and the natural enemy community will barely differ between traditional olive groves (with intermediate levels of perturbation) and organic olive groves (with slightly lower levels of perturbation). Nevertheless, the natural enemy community structure and composition would be expected to differ between managed and abandoned groves, with the latter harboring a richer and more abundant natural enemy community. We also hypothesized that managed groves will host more *B. oleae* individuals.

## 2. Materials and Methods

### 2.1. Study Area

The study was carried out in the municipality of Ahigal de los Aceiteros (Salamanca, Western Spain) (40°52′ N, 6°44′ W). The location of this region, at the Portuguese border, with small villages and limited infrastructure, has led to a continuous process of isolation, depopulation, and farmland abandonment. Conversely, a significant number of the managed plots are transitioning to organic agriculture, with some of them having been organic certified since 2014. Therefore, the agricultural landscape of this region is practically devoid of synthetic pesticides and fertilizers, and structurally, it is a mosaic formed by the combination of managed plots (in many cases with the absence of mechanization and low investment) and abandoned plots in various stages of plant succession, interspersed with fragments of natural vegetation. These remnants of natural vegetation typical of Mediterranean sclerophyllous scrub are formed mainly by *Cytisus* shrublands (*Cytisus multiflorus* (L’Hér.) Sweet, *Cytisus scoparius* (L.) Link), rock rose (*Cistus ladanifer* L.), French lavender (*Lavandula pedunculata* (Mill.) Cav.), and thymes (*Thymus mastichina* (L.) L., *Thymus zygis* subsp. *zygis* Loefl. ex L.). There are also areas of Mediterranean forest, mainly composed of holm oaks (*Quercus ilex* subsp. *ballota* (Desf.) Samp.) and oaks (*Quercus pyrenaica* Willd.), although they coexist with European nettle trees (*Celtis australis* L.) and junipers (*Juniperus oxycedrus* L.), which have great ecological value and persist mainly because of the difficulty of cultivating on the steep slopes of the area, which has considerably limited agricultural practices.

**Figure 1:**
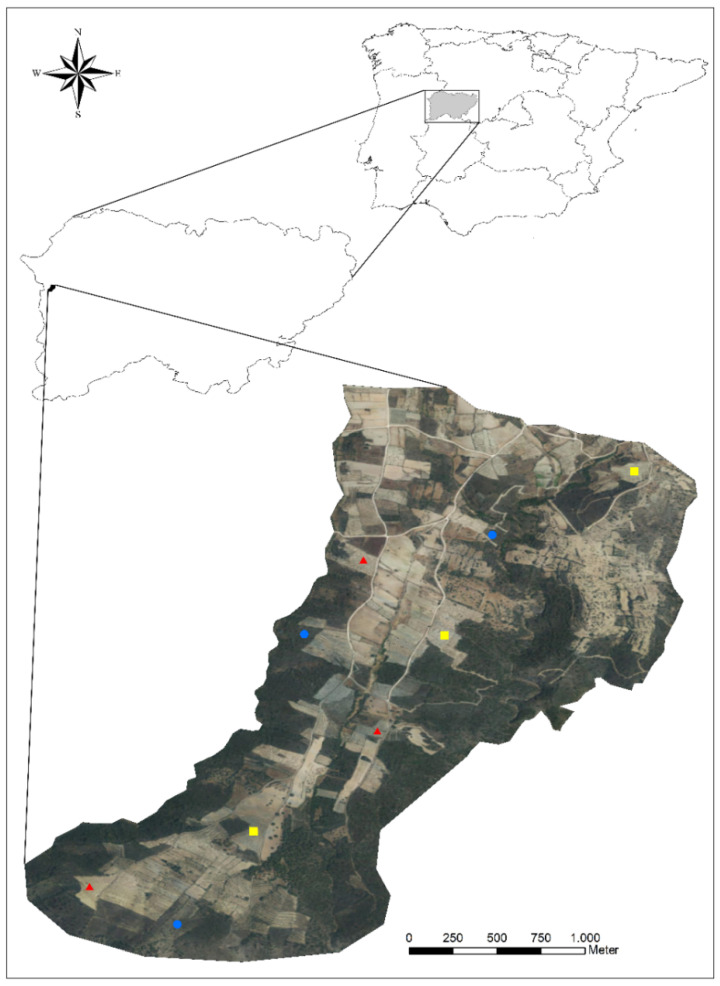
Location of the study area in Salamanca (Western Spain) and the location of the nine olive groves sampled within the study area. Traditional olive groves: yellow squares, organic groves: red triangles, and abandoned groves: blue circles.

The study area comprises 435 ha, its altitude ranging between 405 and 662 m a.s.l. It is located in the transition zone between landscape units 84 (gorges and valleys on the Portuguese border) and 49 (peneplains of Zamora and Salamanca, as well as the foothill of the Montes de León) [38], within the territory of the Arribes del Duero Natural Park (Figure 1). The climate is mild, generally warm, and temperate, with an average annual rainfall of 541 mm and an average annual temperature of 13.7 °C. The predominant soil types are cambisol and leptosol. The study area is bordered by two small streams and in its southwest region by the Águeda River, which runs through a deep canyon. The landscape is dominated by olive groves and, to a lesser extent, by vineyards and almond orchards under a traditional farming system without the application of pesticides and synthetic fertilizers. Olive cultivation is the base of the economic activity within the municipality, with the production and commercialization of extra virgin olive oil and extra virgin organic olive oil as derivative products from different varieties such as *Zorzal de Arribes* (endemic to Arribes del Duero), *Manzanilla Cacerena*, and *Picual*, among others.

### 2.2. Sampling Design

We selected nine olive groves: three organic certified, three traditional, and three abandoned (mean area: 12,822.2 ± 4237.3 m^2^, mean distance to the nearest grove: 564.4 ± 120.4 m). The organic olive groves obtained their certification in 2014; therefore, synthetic pesticides and fertilizers have not been used in these groves at least since 2011. In the traditional category, we included orchards under traditional management that did not have an organic certification but where synthetic pesticides and fertilizers have not been used for at least 10 years, according to the owners. We included them in our study to check for differences between organic-certified orchards and traditional orchards that follow an organic-like type of management but that have not been officially certified as organic and where an occasional and infrequent application of synthetic pesticides or fertilizers could still be performed. Ground cover vegetation was controlled in both systems by mowing once or twice a year, all groves are rainfed, and they have an average production of 12 kilos of olives per tree. Finally, all abandoned groves had been abandoned for at least 15 years.

Sampling was performed from May to October 2018 every seven to eight weeks, starting with the flowering period (early May) and finishing just before the olive harvest (late October), attending to the period of highest arthropod abundance [39] and following the *B. oleae* life cycle [40]. Each sampling took place over six consecutive days, randomly assigning the order in which the groves were sampled. Weather conditions were kept as uniform as possible between the sampling periods, avoiding rainy and windy days.

**Figure 2:**
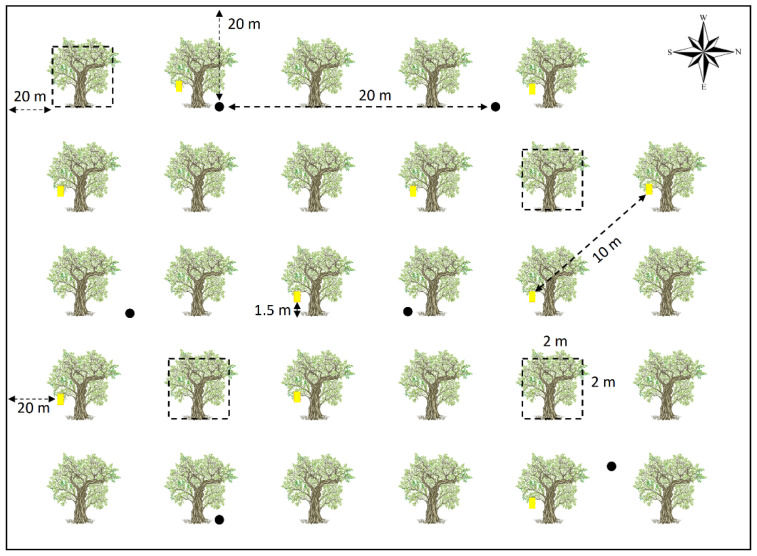
Sampling design. Black circles: pitfall traps; yellow rectangles: sticky traps; empty squares: 2 m × 2 m vacuuming quadrants. Dashed arrows represent the minimum distance between traps or the grove’s edge.

To capture edaphic fauna (spiders, carabids, staphylinids, and ants) six uncovered pitfall traps (9 cm diameter, 12.3 cm depth) were placed in each grove, three under the olive tree canopy and three between rows (54 in total). The traps were filled to a third with a mixture of 70% alcohol and antifreeze (10% ethylene glycol) in a 3:2 ratio (600 mL of alcohol and 400 mL of antifreeze per liter). The traps were also placed 20 m apart from each other and the groves’ edges to reduce trap-to-trap interference and edge effects, and remained in the field for 72 h. To collect hymenopteran parasitoids and vegetation spiders, we randomly selected four trees in each olive grove and vacuumed each tree and the surrounding vegetation in a 2 m × 2 m quadrant for three minutes using a gardener’s leaf-blower (Garland GAS 550 G) [41] modified as a suction machine. For the capture of *B. oleae* specimens, we placed 10 chromatic sticky traps (25 cm × 10 cm) (Koppert Biological Systems—Horiver) in each grove. The traps were hung from the lower branches of the olive trees (at a height of 1.5–2 m above the ground), arranged with a south-facing orientation and separated by a minimum of 10 m from each other, and remained active for 72 h (Figure 2).

All the collected specimens were sorted in the laboratory and identified at the family level. This higher taxa approach (e.g., family taxonomic resolution) was found to be a reliable approach for revealing species richness and compositional patterns [42].

### 2.3. Statistical Analyses

Prior to performing the analyses, we assessed the completeness of the sampling methods for each group (spiders, parasitoids, and natural enemy community (spiders, parasitoids, staphylinids, carabids, and ants)) using the non-parametric Chao1 estimator [43]. Both methods exhibited high levels of completeness (pitfall traps: 92% of the 12 estimated parasitoid families, 96% of the 25 estimated spider families, and 93% of the 41 estimated natural enemy families; vacuuming: 96% of the 23 estimated parasitoid families, 84% of the 19 estimated spider families, and 80% of the 51 estimated natural enemy families).

The effects of the *system* type (organic certified, traditional, and abandoned) on the natural enemy, spider, and parasitoid communities were analyzed with PERMANOVA (*system* and *sampling month* as fixed factors, with 9999 permutations, and “permutation of residuals under a reduced model” as the permutation method) and MDS (multidimensional scaling). Similarity matrices were calculated using Bray–Curtis coefficients with the abundances square-root transformed to reduce the weight of the most dominant families. Considering that the PERMANOVA results (Table 1) revealed no significant differences between the organic-certified and traditional plots, we excluded the latter in the rest of the analyses and focused on studying the differences between organic and abandoned groves.

To test for spatial autocorrelation between the study sites, we performed a Mantel correlogram based on a similarity matrix (Bray–Curtis) and the geographical coordinates of the study sites [44]. The results revealed a significant spatial autocorrelation for the parasitoids and for the spider families Gnaphosidae and Linyphiidae. Therefore, we added spatial correlation structures to these models and compared their AICs to select the best model [40]. We also checked for temporal correlation using the autocorrelation function (ACF), and when we detected significant temporal patterns, we added a correlation structure for a short time series and a variance structure that allowed for different variances for each level of the variable *sampling month* to our models and compared the AICs [45].

We used linear least-squares models, linear mixed models, generalized linear models, and generalized linear mixed models to test the effect of the *system* (organic certified or abandoned), the *sampling month* and the interaction (when significant) (as fixed factors), and the *site* as a random factor (when applicable) on the family richness, abundance, and Shannon’s index values for the natural enemies, spiders, and parasitoids and for the abundance of the most dominant families of the whole natural enemy community (>60 individuals, 17 out of 52 families). The residuals of each model were checked to ensure normality, independence, and homoscedasticity. Additionally, a factorial correspondence analysis (FCA) was carried out to represent the association between these families and the two types of olive groves (abandoned and organic).

To analyze the possible effect of the *system* (organic certified, traditional, and abandoned) on the populations of *B. oleae*, we applied a generalized linear mixed-effects model (GLMM), with the variables *system*, the *sampling month*, and their interaction as fixed factors, and the *site* as a random factor. Since we did not detect any *B. oleae* adults in the two summer sampling periods, we excluded them from the analysis, as the high number of zeros could be a source of error in the analysis. Then, we performed the analysis only with the data from the first and last sampling periods (May and October, respectively).

For the analyses, the statistical packages PRIMER v6 (PERMANOVA, MDS) (PRIMER-E Ltd., Plymouth, UK) [46], R 3.6.2 (linear least-squares models, linear mixed models, generalized linear models, generalized linear mixed models, and zero-inflated models) [47], and XlStat 2014 (factorial correspondence analysis) [48] were used.

## 3. Results

A total of 13,300 arthropods belonging to the focal groups were collected—1730 spiders (26 families), 2125 parasitoids (22 families), 27 carabids, 255 staphylinids, 8483 ants and, and 680 *B. oleae* individuals.

**Figure 3:**
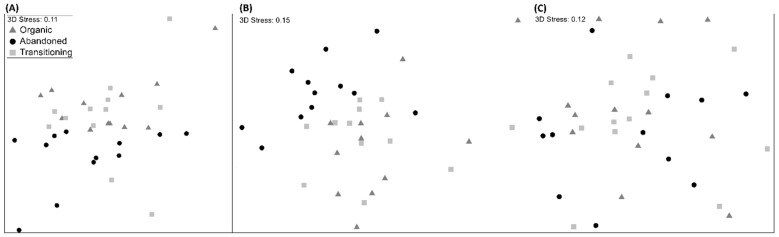
MDS of the natural enemy (**A**), spider (**B**), and parasitoid (**C**) communities sampled (Bray–Curtis index, square-root transformed abundances). Triangles: organic groves; circles: abandoned groves; squares: traditional groves.

The PERMANOVA revealed a significant effect of the *system* on the natural enemy and spider communities (pseudo-F = 2.312, *p* = 0.0001 and pseudo-F = 3.052, *p* = 0.0001, respectively) but none for the parasitoid community (pseudo-F = 0.7927, *p* = 0.702); these differences occurred only between the managed and abandoned systems but not between the organic and traditional plots (Table 1). These results were also noticeable in the MDS, where, for the natural enemy and spider communities, the abandoned groves were clearly separated from the managed groves, which was not the case for the parasitoid community (Figure 3).

When considering the whole natural enemy community, we did not find differences in richness, abundance, or diversity between organic and abandoned groves (Table 2). Nevertheless, the models showed that spiders were significantly affected by the *system*, with the abandoned plots harboring richer and more abundant communities, although we also found a marginally significantly higher spider diversity in the organic groves toward the end of the sampling period. Parasitoids were partly affected by the *system*, showing no differences in richness or abundance, but their diversity was significantly higher in the abandoned plots. This difference decreased throughout the sampling period, and at the end of the season, organic plots harbored a higher parasitoid diversity (Table 2), showing a trend similar to that of the spiders. 

**Figure 4:**
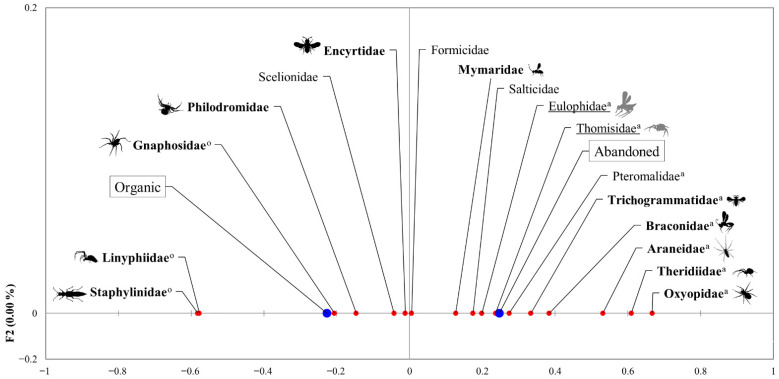
Factorial correspondence analysis was performed on the abundance of the most relevant families of natural enemies on each type of *system* (organic and abandoned). Families in bold with black drawings were those significantly affected by the variable *system*, whereas underlined families with grey drawings were marginally affected by this variable, according to the results obtained from the linear models fitted on the abundances of the most dominant families. Red dot: principal coordinate value for each family in the first axis of the FCA; blue dot: principal coordinate value for each farming system in the first axis of the FCA. ^o^ Significant association with the organic groves in the correspondence analysis; ^a^ significant association with the abandoned groves in the correspondence analysis.

The results of the linear models applied to the most abundant families showed different responses to the variable *system* across taxa. Nevertheless, we found a general tendency of natural enemy families to associate with abandoned groves (nine out of seventeen), with only four families being more abundant in the organic groves (Gnaphosidae, Linyphiidae, Philodromidae, and Staphylinidae) (Table 3). Four families were not associated with either type of *system* (Formicidae, Pteromalidae, Salticidae, and Scelionidae). These results were consistent with those of the correspondence analysis (Figure 4), except for four families. Mymaridae and Encyrtidae were more abundant in the abandoned groves according to the models, but they were not associated with these groves in the correspondence analysis; the contrary applied to the family Pteromalidae, which was associated with the abandoned groves in the correspondence analysis but not according to the generalized linear mixed model. The results of the model for the family Philodromidae revealed a significantly higher abundance in the organic groves that was not supported by the correspondence analysis (Figure 4). In the case of the main olive pest, *B. oleae*, the managed groves harbored significantly higher abundance than the abandoned groves in October (Table 3). In May, the *B. oleae* abundance was too low to detect differences between systems (105 versus 575 individuals in October). We did not capture any adults during the summer sampling.

## 4. Discussion

In accordance with our hypothesis, the structure of the natural enemy community differed between the abandoned and organic olive groves, except when considering parasitoid wasps alone. Parasitoid wasps were the most mobile group in the study, and they tend to be more affected by variables at a landscape scale [49,50,51]. They were most likely moving along patches adjacent to the groves in the matrix, using the multiple resources in abandoned groves and semi-natural habitats for foraging, refuge, and alternative hosts [52], and spilling over to managed groves, mostly searching for hosts [53]. In agreement with our hypothesis, we found no differences between organic and traditional groves. The landscape complexity of our study area would explain the lack of effect of the farming system (according to the *intermediate landscape complexity hypothesis*) [9], where local extensification measures, such as organic farming, are expected to have little effect on species richness [8]. Moreover, Bruggisser et al. [10] suggested that “the biodiversity benefits of organic farming in annual cropping systems may not hold for perennial crops, particularly if the use of pesticides is minimal,” based on the *intermediate disturbance hypothesis* [11]. Nevertheless, the similarity between organic and traditional groves also indicated that traditional agriculture, with a very occasional input of synthetic pesticides and fertilizers, may be able to maintain similar levels of natural enemy diversity as organic agriculture. Therefore, our results, in agreement with those of other studies, highlighted traditional agriculture as high-nature-value farming systems that supported high biodiversity in agricultural landscapes [54,55]. As we expected, the main olive pest, namely, *B. oleae*, was associated with the managed groves. In the abandoned groves, given the lack of management (especially pruning), olive trees produced less fruit; therefore, olive availability for *B. oleae* oviposition was scarce, and few adults developed in unmanaged habitats. In fact, olive production was so low that the *B. oleae* adults that we found in the abandoned groves may have dispersed from the managed groves. The abandoned groves would not act as reservoirs of *B. oleae*; they may even be sink habitats (i.e., resource-poor habitats where the death rate exceeds the birth rate and the populations are maintained, in the long term, by immigration [56]) because of the higher rate of natural enemies, functioning similarly to trap crops, which attract pests and induce a higher rate of pest mortality (e.g., through reduced larval survival or suppression by natural enemies) [57,58].

Surprisingly, we did not find differences in richness, abundance, or diversity for the whole natural enemy community between organic and abandoned olive groves, even though the structure of the communities tended to diverge. Both organic and abandoned groves would provide valuable and diverse microhabitats that harbor different natural enemy communities but be similarly rich and diverse given their high niche availability [15,59]. Abandoned groves, with an intermediate structure between the more open habitat of managed groves, and the closer one of the Mediterranean shrubland and forest, with structurally dense vegetation, maintain diverse communities that are different from managed groves and probably also from the natural habitats that surround them (see [22] for an example with spiders in European grasslands). Consequently, abandoned groves may increase landscape complexity, as well as microhabitat and resource availability, in agreement with other studies in different ecosystems (e.g., [59] for fruit orchards and [22] for grasslands; see [18] for a review).

Focusing on the most important groups of natural enemies considered in this study, namely, spiders and parasitoids, we found that the abandoned groves harbored richer and more abundant spider communities and higher parasitoid diversity. The structurally more complex vegetation of abandoned groves provided a wider range of microhabitats and especially more resources for feeding and refuge, which may have favored these groups. Several studies have shown that spiders are constrained by different habitat features at a local scale (e.g., [60,61]), favoring habitats with more complex vegetation architecture [62,63,64,65] and increased prey availability [66,67]. Therefore, the characteristics of the abandoned groves allowed them to sustain richer and more abundant spider communities. Concerning parasitoid wasps, the abandoned groves had a higher habitat complexity, which was found to enhance natural enemies in olive groves, but with little effects in parasitoid abundance [68]. Not only do these groves provide more resources (i.e., floral resources, overwintering sites, alternative hosts) that favor parasitoids [69] but also this may reduce negative interactions (such as intraguild predation, competition, or hyperparasitism) [70,71], which result in a more even community with higher diversity values.

Regarding community changes throughout the season, we detected a change of trend in the diversity observed in the two systems, with the organic groves harboring a higher diversity of both spiders and parasitoids than the abandoned groves toward the end of the sampling period. In the studied areas, farmers managed the natural vegetation cover in productive groves through conservation tillage (i.e., a form of non-intensive tillage that leaves at least 30% of the previous cover’s residue on the surface [72]) in July. It was shown that rapid perturbations, such as tillage, predominantly affect dominant species [73], reducing competition over hosts/prey and other resources. Therefore, the organic groves harbored a slightly higher richness of spiders and parasitoids in October, which, when combined with the lower abundance found in this month, resulted in a significantly higher diversity. In fact, conservation tillage was shown to favor predators and parasitoids in simple and complex landscapes [74] and to increase predator diversity and evenness [75]. Our results are in accordance with those of previous studies that highlight that mild disturbances of traditional agroecosystems can favor biodiversity [19,21,54,55,59].

According to our expectations, the abandoned groves achieved a higher abundance of the most dominant families of natural enemies in general but with clear differences between taxa. Five out of seven parasitoid families were more abundant in the abandoned groves, with none associating with the organic groves. Parasitoids belong to a high trophic level, and they are especially sensitive to environmental change and agricultural disturbances [76,77]; thus, we would have expected to find differences in the community composition or shifts in richness or abundance between these two systems. A possible explanation is that an evaluation at the family level may not provide sufficient resolution to detect these differences [78]. However, when studying the dominant families separately, we do see a clear association of these families with the abandoned olive groves, again emphasizing their higher abundance of food and shelter resources that would favor parasitoids [69], and also their heightened chemical complexity, which may attract a greater diversity of parasitoids [24,79].

For the spiders, we found mixed results across families. The families Araneidae and Theridiidae (space and orb-web weavers, respectively [62,80]) and Oxyopidae and Thomisidae (active hunters and ambush hunters, respectively, mostly on the vegetation, with the latter especially on flowers [62,80]) are more abundant in the abandoned groves. As previously mentioned, these families probably favored the higher plant structural complexity of the abandoned groves, which provided more anchoring points for the webs of theridids and araneids and foraging, as well as refuge resources for oxyopids and thomisids, along with a variety of locations for ambushing prey for the latter [62,63,67]. On the other hand, the families Gnaphosidae, Linyphiidae, and Philodromidae are more abundant in the organic groves. Gnaphosids are ground hunters, which favor open habitats, such as those of the organic groves [64,81,82]. Philodromids are active hunters either on the ground or the vegetation, and linyphiids include sheet-web weavers and also active hunters, both groups with ground and vegetation species. These two families may be more abundant in the organic groves because of less competition with species from other families that need more structurally complex habitats to flourish. Another possible explanation is that organic groves have filtered some ground-dwelling species of these families that occur mainly in open habitats [63,83] and that are dominating the spider community in these groves.

Staphylinids were more abundant in the organic groves, in contrast with the results of Baloj and Markó [84,85], who found more abundant staphylinid communities in abandoned apple orchards and vineyards than in productive fields (conventional and IPM and conventional and organic, respectively). This result may indicate that the community composition of staphylinids in our study area was dominated by generalist and open-habitat species that favored the agricultural management of the organic groves [86].

Four families were not associated with either system. In the case of scelionids and pteromalids, some species may favor the organic groves, masking the possible differences between organic and abandoned groves (e.g., parasitoids of ground beetles, which are more abundant in the organic groves, or parasitoids of olive pests). We also did not find any differences in ant abundance. Since we did not sort them into species or functional groups, we cannot assess the structure of the ant community. Based on the few studies that compare ant diversity in abandoned and managed agricultural fields ([87] in orchards and [88] in cereal fields), we can hypothesize that abandoned groves may harbor a different, more diverse ant community because of their higher niche and different resource availability, with organic groves harboring fewer species, but they are exploiting the resources successfully, resulting in similar abundance levels. Finally, the family Salticidae includes some species adapted to hunting on the ground, while others hunt on the vegetation [80]; thus, open-habitat species may be more abundant in organic groves, and the more complex vegetation of abandoned groves may sustain more individuals of species that hunt on the vegetation, although they tend to prefer widely spaced over dense structures [89].

Although arthropod interannual population variability may result in biased conclusions [90], short-term studies provide valuable results that are useful for making local comparisons [91], and most studies conducted with arthropods in agroecosystems have one-year sampling periods. In any case, our results should be interpreted with caution due to their dependence on spatial and temporal context.

## 5. Conclusions

Our study provided the first evidence of the effects of olive grove abandonment on *B. oleae* and different groups of natural enemies in traditional agroecosystems and contributed to increasing the knowledge of these effects on woody crops, which are clearly underrepresented in the literature, even when considering the limitations derived by the fact that our sampling period was restricted to one year. The studied traditional olive groves may be able to sustain a different natural enemy community (but equally rich and diverse) than that of the more structurally complex abandoned groves, emphasizing the role that traditional olive groves have in conserving biodiversity in agricultural landscapes. Abandoned groves could constitute landscape elements of high importance that provide a wide variety of microhabitats and resources that are exploited by natural enemies but not by the olive fruit fly and, therefore, may not act as reservoirs for this pest in our study area. The presence of both systems, traditional and abandoned groves, at a landscape scale provides complimentary habitats that promote diverse communities of some beneficial groups, such as spiders. However, some management measures should be carried out in the abandoned groves to halt plant succession, preventing them from becoming Mediterranean scrubland or forest, similar to the one surrounding them, which would result in the loss of landscape-scale heterogeneity and biodiversity. A greater priority is to stop the abandonment of traditional olive groves that are still in use. The high biodiversity of these groves depends on their traditional management, and further abandonment would translate not only into the loss of the aesthetic and cultural values associated with these agroecosystems but also the possibly irretrievable loss of the biodiversity that they harbor. For this reason, the measures aimed at reducing the depopulation of rural areas, where these traditional systems occur, are especially important to stop the abandonment process. Organic agriculture—with the associated increase in the price of olive oil, which results in higher profits for farmers—and agricultural tourism are measures that were shown to be very effective in halting this process in our study area. More studies are needed to disentangle the effects of olive grove abandonment on the associated arthropod communities, especially in different regions and with longer sampling periods.

## Figures and Tables

**Table 1:** Results of the PERMANOVA for the variables *system* and *sampling month* (full model results and pairwise comparisons among the three systems).

Response Variable	Explanatory Variables	d.f.	Pseudo-F	*p*-Value
Natural enemies	*System*	2	2.3117	0.0001
	*Sampling month*	3	8.9107	0.0001
Spiders	*System*	2	3.0515	0.0001
	*Sampling month*	3	6.2039	0.0001
Parasitoids	*System*	2	0.7927	0.7016
	*Sampling month*	3	5.3291	0.0001
**Pairwise Comparisons**			**Pseudo-t**	***p*-Value**
Natural enemies	Abandoned, organic		1.7466	0.0001
	Abandoned, traditional		1.7311	0.0002
	Organic, traditional		0.9225	0.6299
Spiders	Abandoned, organic		2.0979	0.0001
	Abandoned, traditional		2.0336	0.0001
	Organic, traditional		0.7412	0.8070
Parasitoids	Abandoned, organic		0.6894	0.8338
	Abandoned, traditional		1.0252	0.4277
	Organic, traditional		0.9476	0.5434

**Table 2:** Results of the different models for the richness, abundance, and diversity (Shannon index (H)) of natural enemies, spiders, and parasitoids. Estimates, standard errors, test statistics, *p*-values, and significance levels (ns > 0.1, · < 0.1, * < 0.05, and *** < 0.001) for the intercept and the explanatory variable *system*, as well as the interaction between the variables *system* and the *sampling month* (when significant), are provided. The complete results, including the explanatory variable *sampling month*, are given in Table S1.

Response Variable	Explanatory Variable	Value/Estimate	Std. Error	*t*-Value/*z*-Value	*p*-Value	*
Natural enemy family richness ^a^	Intercept	20.667	1.208	17.114	<0.001	***
	*System* (organic)	−0.667	1.080	−0.617	0.544	ns
Natural enemy abundance ^a^	Intercept	247.081	33.023	7.482	<0.001	***
	*System* (organic)	4.5057	31.315	0.144	0.887	ns
Natural enemy diversity (H) ^a^	Intercept	1.557	0.159	9.789	<0.001	***
	*System* (organic)	−0.155	0.142	−1.087	0.291	ns
Spider family richness (square root) ^a^	Intercept	2.838	0.094	30.134	<0.001	***
	*System* (organic)	−0.157	0.072	−2.161	0.044	*
Spider abundance ^b^	Intercept	3.200	0.126	25.352	<0.001	***
	*System* (organic)	−0.346	0.136	−2.555	0.011	*
Spider diversity (H) ^a^	Intercept	1.913	0.140	13.675	<0.001	***
	*System* (organic)	0.075	0.183	0.409	0.689	ns
	*Sampling month* (June): *system* (organic)	−0.135	0.258	−0.524	0.608	ns
	*Sampling month* (August): *system* (organic)	0.423	0.205	2.059	0.056	·
	*Sampling month* (October): *system* (organic)	0.467	0.247	1.894	0.077	·
Parasitoid family richness ^c^	Intercept	2.368	0.107	22.136	<0.001	***
	*System* (organic)	−0.034	0.099	−0.347	0.732	ns
Parasitoid abundance ^d^	Intercept	48.917	8.685	5.632	<0.001	***
	*System* (organic)	−5.833	10.328	−0.565	0.602	ns
Parasitoid diversity ^a^	Intercept	2.144	0.130	16.445	<0.001	***
	*System* (organic)	−0.473	0.184	−2.568	0.021	*
	*Sampling month* (June): *system* (organic)	0.421	0.200	2.103	0.052	·
	*Sampling month* (August): *system* (organic)	0.108	0.277	0.389	0.702	ns
	*Sampling month* (October): *system* (organic)	0.701	0.303	2.317	0.034	*

^a^ GLS; ^b^ GLMM with a Poisson distribution; ^c^ quasi-GLM model; ^d^ LME.

**Table 3:** Results of the different linear models for the abundance of the most dominant families. Estimates, standard errors, test statistics, *p*-values, and significance levels (ns > 0.1, · < 0.1, * < 0.05, ** < 0.01, and *** < 0.001) for the intercept and the explanatory variable *system*, as well as the interaction between the variables *system* and the *sampling month* (when significant), are provided. No results are shown for Formicidae because the variable *system* did not remain in the optimal model. The complete results, including the explanatory variable *sampling month*, are given in Table S2.

Response Variable	Explanatory Variable	Value/Estimate	Std. Error	*t*-Value/*z*-Value	*p*-Value	*
Araneidae abundance ^a^	Intercept	1.680	0.244	6.875	<0.001	***
	*System* (organic)	−1.069	0.244	−4.376	<0.001	***
Gnaphosidae abundance ^a^	Intercept	0.784	0.491	1.597	0.110	ns
	System (organic)	0.584	0.650	0.899	0.369	ns
	*Sampling month* (June): *system* (organic)	−0.657	0.557	−1.179	0.238	ns
	*Sampling month* (August): *system* (organic)	0.208	0.601	0.346	0.730	ns
	*Sampling month* (October): *system* (organic)	1.594	0.865	1.843	0.065	·
Linyphiidae abundance ^a^	Intercept	0.654	0.474	1.379	0.168	ns
	System (organic)	0.758	0.599	1.266	0.205	ns
	*Sampling month* (June): *system* (organic)	1.391	0.608	2.287	0.022	*
	*Sampling month* (August): *system* (organic)	−5.11E-05	0.625	0	0.999	ns
	*Sampling month* (October): *system* (organic)	−0.074	0.688	−0.108	0.914	ns
Oxyopidae abundance ^a^	Intercept	0.834	0.388	2.152	0.031	*
	System (organic)	−1.946	1.068	−1.823	0.068	·
	*Sampling month* (June): *system* (organic)	−0.251	1.500	−0.168	0.867	ns
	*Sampling month* (August): *system* (organic)	−1.571	0.538	−2.92	0.004	**
	*Sampling month* (October): *system* (organic)	−2.683	0.624	−4.301	<0.001	***
Philodromidae abundance (square root) ^b^	Intercept	0.334	0.378	0.884	0.388	ns
	*System* (organic)	0.576	0.114	5.058	<0.001	***
Salticidae abundance ^b^	Intercept	2.000	0.645	3.098	0.006	**
	*System* (organic)	−0.667	0.577	−1.155	0.263	ns
Theridiidae abundance ^a^	Intercept	0.245	0.466	0.525	0.600	ns
	*System* (organic)	−1.287	0.310	−4.15	<0.001	***
Thomisidae abundance ^a^	Intercept	0.835	0.390	2.141	0.032	*
	*System* (organic)	−0.002	0.550	−0.004	0.997	ns
	*Sampling month* (June): *system* (organic)	0.167	0.673	0.248	0.804	ns
	*Sampling month* (August): *system* (organic)	−1.447	0.770	−1.879	0.060	·
	*Sampling month* (October): *system* (organic)	−0.442	0.683	−0.646	0.518	ns
Braconidae abundance (square root) ^b^	Intercept	2.943	0.529	5.567	<0.001	***
	System (organic)	−2.000	0.748	−2.675	0.017	*
	*Sampling month* (June): *system* (organic)	1.576	0.835	1.889	0.077	·
	*Sampling month* (August): *system* (organic)	1.805	0.857	2.105	0.051	·
	*Sampling month* (October): *system* (organic)	2.667	0.933	2.858	0.011	*
Encyrtidae abundance ^a^	Intercept	1.194	0.334	3.576	<0.001	***
	*System* (organic)	−1.625	0.789	−2.061	0.039	*
	*Sampling month* (June): *system* (organic)	2.197	0.837	2.625	0.007	**
	*Sampling month* (August): *system* (organic)	−0.056	0.876	−0.064	0.949	ns
	*Sampling month* (October): *system* (organic)	3.091	0.848	3.644	<0.001	***
Eulophidae abundance ^a^	Intercept	1.814	0.200	9.058	<0.001	***
	*System* (organic)	−0.321	0.173	−1.853	0.064	·
Mymaridae abundance ^b^	Intercept	1.843	0.197	9.337	<0.001	***
	*System* (organic)	−0.530	0.175	−3.034	0.007	**
Pteromalidae abundance ^a^	Intercept	0.668	0.530	1.259	0.208	ns
	*System* (organic)	−0.372	0.664	−0.561	0.575	ns
Scelionidae abundance ^a^	Intercept	2.557	0.133	19.257	<0.001	***
	*System* (organic)	0.169	0.120	1.401	0.161	ns
Trichogrammatidae abundance ^b^	Intercept	1.426	0.233	6.126	<0.001	***
	*System* (organic)	−0.600	0.228	−2.634	0.016	*
Staphylinidae abundance ^a^	Intercept	1.936	0.353	5.479	<0.001	***
	*System* (organic)	1.405	0.469	2.998	0.003	**
*Bactrocera oleae* abundance ^a^	Intercept	1.662	0.566	2.938	0.003	**
	*System* (traditional)	0.346	0.788	0.439	0.660	ns
	*System* (organic)	0.859	0.775	1.108	0.268	ns
	*Sampling month* (October): *system* (traditional)	1.820	0.302	6.027	<0.001	***
	*Sampling month* (October): *system* (organic)	1.531	0.263	5.817	<0.001	***

^a^ GLMM with a Poisson distribution; ^b^ GLS.

## Data Availability

The data presented in this study are available as Supplementary Materials (Supplementary Data Matrix).

## Supplementary Materials

The following are available online at https://www.mdpi.com/article/10.3390/insects13010048/s1, Table S1: Complete results of the different models for the richness, abundance, and diversity (Shannon index (H)) of natural enemies, spiders, and parasitoids; Table S2: Complete results of the different models for the abundance of the most dominant families and *B. oleae*; Supplementary Data Matrix: Our complete data set.
