# Supplementary material for: Effect of Organic Farming and Agricultural Abandonment on Beneficial Arthropod Communities Associated with Olive Groves in Western Spain: Implications for Bactrocera oleae Management
Source: Insects. 2022 Jan 1;13(1):48. doi: 10.3390/insects13010048 (PMC8778029; doi:10.3390/insects13010048)
Supplement: Supplementary file 1 [file insects-13-00048-s001.zip › Suplementary Material Table S1 and S2.pdf]

Table S1. Complete results of the different models for the richness, abundance, and diversity (Shannon index (H)) of natural enemies, spiders, and parasitoids. Estimates, standard errors, test statistics, p-values, and significance levels (ns > 0.1, · < 0.1, * < 0.05, ** < 0.01, and *** < 0.001) for the intercept and the explanatory variables s*ystem*, the *sampling month*, and their interaction (when significant) are provided. In the first column, the type of model (GLS, generalized least squares model; LME, linear mixed model; GLM, generalized linear model; GLMM, generalized linear mixed model), the transformation applied on the explanatory variable (if any), and variance structure added to the model (if any) are given.

Model	Response variable	Explanatory variable	Value / Estimate	Std. error	t-value / z-value	p-value	*
GLS	Natural enemy richness	Intercept	20.667	1.208	17.114	<0.001	***
		System (Organic)	-0.667	1.080	-0.617	0.544	ns
		Sampling month (June)	2.667	1.528	1.746	0.097	·
		Sampling month (August)	0.833	1.528	0.546	0.592	ns
		Sampling month (October)	1.167	1.528	0.764	0.454	ns
GLS (varIdent (form=~1| *Sampling month*)	Natural enemy abundance	Intercept	247.081	33.023	7.482	<0.001	***
		System (Organic)	4.506	31.315	0.143	0.887	ns
		Sampling month (June)	371.000	133.427	2.781	0.012	*
		Sampling month (August)	152.833	54.330	2.813	0.011	*
		Sampling month (October)	-83.000	35.618	-2.330	0.031	*
GLS	Natural enemy diversity (H)	Intercept	1.557	0.159	9.789	<0.001	***
		System (Organic)	-0.155	0.142	-1.087	0.291	ns
		Sampling month (June)	-0.238	0.201	-1.184	0.251	ns
		Sampling month (August)	-0.072	0.201	-0.357	0.725	ns
		Sampling month (October)	0.747	0.201	3.713	0.002	**
GLS (square root) (varIdent (form=~1| *Sampling month*)	Spider richness	Intercept	2.838	0.094	30.134	<0.001	***
		System (Organic)	-0.157	0.072	-2.161	0.044	*
		Sampling month (June)	0.340	0.117	2.905	0.009	**
		Sampling month (August)	0.678	0.101	6.687	<0.001	***
		Sampling month (October)	0.499	0.132	3.773	0.001	**
GLMM Poisson	Spider abundance	Intercept	3.200	0.126	25.352	<0.001	***
		System (Organic)	-0.346	0.136	-2.555	0.011	*
		Sampling month (June)	0.822	0.106	7.73	<0.001	***
		Sampling month (August)	1.304	0.100	13.046	<0.001	***
		Sampling month (October)	0.958	0.104	9.185	< 0.001	***
GLS (varIdent (form=~1| *Sampling month*)	Spider diversity (H)	Intercept	1.913	0.140	13.675	<0.001	***
		System (Organic)	0.075	0.183	0.409	0.688	ns
		Sampling month (June)	-0.106	0.145	-0.729	0.477	ns
		Sampling month (August)	-0.123	0.174	-0.707	0.490	ns
		Sampling month (October)	-0.217	0.198	-1.096	0.289	ns
		Sampling month (June) : *System* (Organic)	-0.135	0.258	-0.524	0.608	ns
		Sampling month (August) : *System* (Organic)	0.423	0.205	2.059	0.056	·
		Sampling month (October) : *System* (Organic)	0.467	0.247	1.894	0.077	·
	Pairwise	abandoned - organic, may == 0	0.217	0.198	1.096	0.732	ns
		abandoned - organic, june == 0	0.352	0.166	2.123	0.283	ns
		abandoned - organic, august == 0	-0.206	0.055	-3.753	0.062	·
		abandoned - organic, october == 0	-0.250	0.147	-1.699	0.435	ns
Quasi-GLM	Parasitoid richness	Intercept	2.368	0.107	22.136	<0.001	***
		System (Organic)	-0.034	0.099	-0.347	0.732	ns
		Sampling month (June)	0.105	0.131	0.803	0.432	ns
		Sampling month (August)	-0.272	0.145	-1.878	0.076	·
		Sampling month (October)	-0.211	0.142	-1.484	0.154	ns

LME	Parasitoid abundance	Intercept	48.917	8.685	5.632	<0.001	***
		System (Organic)	-5.833	10.328	-0.565	0.602	ns
		Sampling month (June)	51.833	7.676	6.753	<0.001	***
		Sampling month (August)	7.833	7.676	1.021	0.324	ns
		Sampling month (October)	-4.000	7.676	-0.521	0.619	ns
GLS (varIdent (form=~1| *Sampling month*)	Parasitoid diversity	Intercept	2.144	0.130	16.445	<0.001	***
		System (Organic)	-0.473	0.184	-2.568	0.021	*
		Sampling month (June)	-0.320	0.141	-2.265	0.038	*
		Sampling month (August)	-0.826	0.196	-4.214	<0.001	***
		Sampling month (October)	-0.737	0.214	-3.442	0.003	**
		Sampling month (June) : *System* (Organic)	0.421	0.200	2.103	0.052	·
		Sampling month (August) : *System* (Organic)	0.108	0.277	0.389	0.702	ns
		Sampling month (October) : *System* (Organic)	0.701	0.303	2.317	0.034	*
	Pairwise	abandoned - organic, may == 0	0.473	0.184	2.568	0.182	ns
		abandoned - organic, june == 0	0.053	0.078	0.680	0.924	ns
		abandoned - organic, august == 0	0.366	0.207	1.768	0.403	ns
		abandoned - organic, october == 0	-0.228	0.240	-0.948	0.809	ns

Table S2. Complete results of the different models for the abundance of the most dominant families and *Bactrocera oleae*. Estimates, standard errors, test statistics, p-values, and significance levels (ns > 0.1, · < 0.1, * < 0.05, ** < 0.01, and *** < 0.001) for the intercept and the explanatory variables *system*, the *sampling month*, and their interaction (when significant) are provided. In the first column the type of model (GLS, generalized least squares model; LME, linear mixed model; GLM, generalized linear model; GLMM, generalized linear mixed model), the transformation applied on the explanatory variable (if any), and the correlation or variance structure added to the model (if any) are given.

Model	Response variable	Explanatory variable	Value / Estimate	Std. error	t-value / z-value	p-value	*
GLMM Poisson	Araneidae abundance	Intercept	1.680	0.244	6.875	<0.001	***
		System (Organic)	-1.069	0.244	-4.376	<0.001	***
		Sampling month (June)	0.310	0.280	1.107	0.268	ns
		Sampling month (August)	0.879	0.253	3.474	<0.001	***
		Sampling month (October)	0.598	0.265	2.257	0.024	*
GLMM Poisson	Gnaphosidae abundance	Intercept	0.784	0.491	1.597	0.110	ns
		System (Organic)	0.584	0.650	0.899	0.369	ns
		Sampling month (June)	0.865	0.417	2.076	0.038	*
		Sampling month (August)	0.118	0.480	0.245	0.806	ns
		Sampling month (October)	-1.386	0.782	-1.774	0.076	·
		Sampling month (June) : *System* (Organic)	-0.657	0.557	-1.179	0.238	ns
		Sampling month (August) : *System* (Organic)	0.208	0.601	0.346	0.730	ns
		Sampling month (October) : *System* (Organic)	1.594	0.865	1.843	0.065	·
	Pairwise	abandoned - organic, may == 0	-0.584	0.650	-0.899	0.774	ns
		abandoned - organic, june == 0	0.073	0.581	0.125	1	ns
		abandoned - organic, august == 0	-0.792	0.623	-1.272	0.512	ns
		abandoned - organic, october == 0	-2.178	0.880	-2.474	0.045	*
GLMM Poisson	Linyphiidae abundance	Intercept	0.654	0.474	1.379	0.168	ns
		System (Organic)	0.758	0.599	1.266	0.205	ns
		Sampling month (June)	0.288	0.535	0.538	0.590	ns
		Sampling month (August)	0.406	0.522	0.777	0.437	ns

		Sampling month (October)	0.00009	0.571	0	0.999	ns
		Sampling month (June) : *System* (Organic)	1.391	0.608	2.287	0.022	*
		Sampling month (August) : *System* (Organic)	-0.00005	0.625	0	0.999	ns
		Sampling month (October) : *System* (Organic)	-0.074	0.688	-0.108	0.914	ns
	Pairwise	abandoned - organic, may == 0	-0.758	0.599	-1.266	0.548	ns
		abandoned - organic, june == 0	-2.149	0.510	-4.216	<0.001	***
		abandoned - organic, august == 0	-0.758	0.529	-1.432	0.435	ns
		abandoned - organic, october == 0	-0.684	0.603	-1.134	0.644	ns
GLMM Poisson	Oxyopidae abundance	Intercept	0.834	0.388	2.152	0.031	*
		System (Organic)	-1.946	1.068	-1.823	0.068	·
		Sampling month (June)	2.929	0.387	7.561	<0.001	***
		Sampling month (August)	2.565	0.392	6.548	<0.001	***
		Sampling month (October)	0.255	0.518	0.492	0.623	ns
		Sampling month (June) : *System* (Organic)	-0.251	1.500	-0.168	0.867	ns
		Sampling month (August) : *System* (Organic)	-1.571	0.538	-2.920	0.004	**
		Sampling month (October) : *System* (Organic)	-2.683	0.624	-4.301	<0.001	***
	Pairwise	abandoned - organic, may == 0	-0.255	0.518	-0.492	0.979	ns
		abandoned - organic, june == 0	-0.004	1.418	-0.003	1	ns
		abandoned - organic, august == 0	1.316	0.227	5.806	<0.001	***
		abandoned - organic, october == 0	2.428	0.389	6.249	<0.001	***
GLS (square root) (varIdent (form=~1| *Sampling month*)	Philodromidae abundance	Intercept	0.334	0.378	0.884	0.388	ns
		System (Organic)	0.576	0.114	5.058	<0.001	***
		Sampling month (June)	0.813	0.380	2.141	0.046	*
		Sampling month (August)	3.079	0.415	7.423	<0.001	***
		Sampling month (October)	2.460	0.401	6.133	<0.001	***
GLS	Salticidae abundance	Intercept	2.000	0.645	3.098	0.006	**
		System (Organic)	-0.667	0.577	-1.155	0.263	ns
		Sampling month (June)	-0.333	0.816	-0.408	0.688	ns
		Sampling month (August)	2.333	0.816	2.858	0.010	*
		Sampling month (October)	1.333	0.816	1.633	0.119	ns
GLMM Poisson	Theridiidae abundance	Intercept	0.245	0.466	0.525	0.600	ns
		System (Organic)	-1.287	0.310	-4.15	<0.001	***
		Sampling month (June)	2.028	0.475	4.274	<0.001	***
		Sampling month (August)	1.609	0.489	3.293	0.0009	***
		Sampling month (October)	1.386	0.499	2.779	0.005	**
GLMM Poisson	Thomisidae abundance	Intercept	0.835	0.390	2.141	0.032	*
		System (Organic)	-0.002	0.550	-0.004	0.997	ns
		Sampling month (June)	0.452	0.483	0.936	0.349	ns
		Sampling month (August)	0.887	0.448	1.979	0.048	*
		Sampling month (October)	0.693	0.462	1.499	0.134	ns
		Sampling month (June) : *System* (Organic)	0.167	0.673	0.248	0.804	ns
		Sampling month (August) : *System* (Organic)	-1.449	0.770	-1.879	0.060	·
		Sampling month (October) : *System* (Organic)	-0.442	0.683	-0.646	0.518	ns
	Paiwise	abandoned - organic, may == 0	0.002	0.550	0.004	1	ns

		abandoned - organic, june == 0	-0.165	0.431	-0.383	0.992	ns
		abandoned - organic, august == 0	1.449	0.571	2.537	0.044	*
		abandoned - organic, october == 0	0.444	0.447	0.992	0.786	ns
GLS (square root) (varIdent (form=~1| *Sampling month*)	Braconidae abundance	Intercept	2.942	0.529	5.567	<0.001	***
		System (Organic)	-2.000	0.748	-2.675	0.017	*
		Sampling month (June)	-0.910	0.590	-1.541	0.143	ns
		Sampling month (August)	-1.943	0.606	-3.205	0.006	**
		Sampling month (October)	-1.894	0.660	-2.870	0.011	*
		Sampling month (June) : *System* (Organic)	1.576	0.835	1.889	0.077	·
		Sampling month (August) : *System* (Organic)	1.805	0.857	2.105	0.051	·
		Sampling month (October) : *System* (Organic)	2.667	0.933	2.858	0.011	*
	Pairwise	abandoned - organic, may == 0	2	0.748	2.675	0.164	ns
		abandoned - organic, june == 0	0.424	0.371	1.142	0.707	ns
		abandoned - organic, august == 0	0.195	0.420	0.465	0.978	ns
		abandoned - organic, october == 0	-0.667	0.559	-1.193	0.680	ns
GLMM Poisson	Encyrtidae abundance	Intercept	1.194	0.334	3.576	<0.001	***
		System (Organic)	-1.625	0.789	-2.061	0.039	*
		Sampling month (June)	0.406	0.407	0.995	0.320	ns
		Sampling month (August)	1.308	0.356	3.679	<0.001	***
		Sampling month (October)	0.00001	0.446	0	0.999	ns
		Sampling month (June) : *System* (Organic)	2.197	0.837	2.625	0.009	**
		Sampling month (August) : *System* (Organic)	-0.056	0.876	-0.064	0.949	ns
		Sampling month (October) : *System* (Organic)	3.091	0.848	3.644	<0.001	***
	Pairwise	abandoned - organic, may == 0	1.625	0.789	2.061	0.146	ns
		abandoned - organic, june == 0	-0.572	0.357	-1.601	0.366	ns
		abandoned - organic, august == 0	1.681	0.440	3.822	<0.001	***
		abandoned - organic, october == 0	-1.466	0.383	-3.829	<0.001	***
GLMM Poisson	Eulophidae abundance	Intercept	1.814	0.200	9.058	<0.001	***
		System (Organic)	-0.321	0.173	-1.853	0.064	·
		Sampling month (June)	1.44	0.196	7.329	<0.001	***
		Sampling month (August)	-0.000007	0.250	0	1	ns
		Sampling month (October)	-0.633	0.300	-2.110	0.035	*
GLS (square root) (varIdent (form=~1| *Sampling month*)	Mymaridae abundance	Intercept	1.843	0.197	9.337	<0.001	***
		System (Organic)	-0.530	0.175	-3.034	0.007	**
		Sampling month (June)	1.802	0.246	7.315	<0.001	***
		Sampling month (August)	-1.041	0.246	-4.226	<0.001	***
		Sampling month (October)	-1.192	0.246	-4.840	<0.001	***
GLMM Poisson	Pteromalidae abundance	Intercept	0.668	0.530	1.259	0.208	ns
		System (Organic)	-0.372	0.664	-0.561	0.575	ns
		Sampling month (June)	0.431	0.352	1.224	0.221	ns
		Sampling month (August)	-1.466	0.633	-2.318	0.021	*
		Sampling month (October)	1.347	0.308	4.381	<0.001	***
GLMM Poisson	Scelionidae abundance	Intercept	2.557	0.133	19.257	<0.001	***

		System (Organic)	0.169	0.120	1.401	0.161	ns
		Sampling month (June)	0.933	0.128	7.289	<0.001	***
		Sampling month (August)	0.895	0.129	6.956	<0.001	***
		Sampling month (October)	0.046	0.152	0.303	0.762	ns
GLS (square root) (correlation = corARMA (c(0.2), p=1, q=0))	Trichogrammatidae abundance	Intercept	1.426	0.233	6.126	<0.001	***
		System (Organic)	-0.600	0.228	-2.634	0.016	*
		Sampling month (June)	1.183	0.290	4.086	<0.001	***
		Sampling month (August)	-0.518	0.284	-1.826	0.084	·
		Sampling month (October)	-0.425	0.285	-1.488	0.153	ns
GLMM Poisson	Staphylinidae abundance	Intercept	1.936	0.353	5.479	<0.001	***
		System (Organic)	1.405	0.469	2.998	0.003	**
		Sampling month (June)	-2.866	0.387	-7.416	<0.001	***
		Sampling month (August)	-3.714	0.581	-6.389	<0.001	***
		Sampling month (October)	-0.669	0.154	-4.342	<0.001	***
GLS (square root) (varIdent (form=~1| *Sampling month*)	Formicidae abundance	Intercept	12.604	0.703	17.916	<0.001	***
		Sampling month (June)	8.312	2.714	3.063	0.006	**
		Sampling month (August)	3.444	1.719	2.003	0.059	·
		Sampling month (October)	-5.598	1.531	-3.656	0.002	**
GLMM Poisson	Bactrocera oleae abundance	Intercept	1.662	0.566	2.938	0.003	**
		System (Traditional)	0.346	0.788	0.439	0.660	ns
		System (Organic)	0.859	0.775	1.108	0.268	ns
		Sampling month (October)	0.406	0.208	1.948	0.052	·
		Month (October) : *System* (Traditional)	1.820	0.302	6.027	<0.001	***
		Month (October) : *System* (Organic)	1.531	0.263	5.817	<0.001	***
